# Transparency of reporting and methodological conduct of prognostic and diagnostic clinical prediction models developed using machine learning in total shoulder arthroplasty: A systematic review and critical appraisal

**DOI:** 10.1177/17585732251412368

**Published:** 2026-01-27

**Authors:** Ajaykumar Shanmugaraj, Bushra Khalid, Mithilesh V Kumar, Kyle N Kunze, Ujash Sheth

**Affiliations:** 1 Faculty of Medicine, 7938 University of British Columbia, Vancouver, BC, Canada; 2 Faculty of Medicine, University of Ottawa, Ottawa, Ontario, Canada; 3 Sports Medicine and Shoulder Institute, 12245 Hospital for Special Surgery, New York, NY, USA; 4 Sunnybrook Orthopaedic Upper Limb (SOUL), Sunnybrook Health Sciences Centre, Division of Orthopaedic Surgery, Department of Surgery, University of Toronto, Toronto, ON, Canada

**Keywords:** Artificial intelligence, supervised machine learning, algorithm, deep learning, predictive modelling, shoulder, arthroplasty, total shoulder arthroplasty

## Abstract

**Background:**

This study aimed to synthesize the applications, performance, and methodological conduct of artificial intelligence (AI) prediction models for total shoulder arthroplasty (TSA) outcomes.

**Methods:**

PUBMED, MEDLINE, EMBASE, and CENTRAL were searched on November 2, 2025 for all articles that utilized AI to develop prognostic or diagnostic prediction models utilizing TSA populations. Methodological quality was assessed using the TRIPOD statement and PROBAST tool.

**Results:**

Twenty-four studies comprising outcomes of 497,365 patients (35.6% female; 69.6 ± 0.9 years) were included. Of these patients, 31.0% underwent rTSA, 29.1% aTSA, and 2.8% hemiarthroplasty. The remaining patients received a mix of aTSA and rTSA, but the exact proportions were not reported in their respective studies. Nine studies applied AI to clinical outcomes (AUC 0.85, 0.65–0.96), seven to adverse events (AUC 0.73, 0.52–0.92), and six to resource utilization (AUC 0.78, 0.59–0.91). All twelve studies comparing AI to traditional regression reported that AI models demonstrated superior performance. The need and caution for external validation was reported in 15 studies (62.5%). The mean TRIPOD adherence was 11.6 items (range 9–15); 19 studies (82.3%) met >50% of criteria, and 6 (26.1%) met >66%. PROBAST rated 16 studies (66.7%) at high risk of bias.

**Conclusion:**

AI prediction models in TSA show poor methodology, especially in calibration, sample size, missing data, and validation, warranting cautious interpretation and clearer direction for future research.

**Level of evidence:** IV, systematic review of level I-IV studies

## Introduction

The utilization of artificial intelligence (AI) to develop both prognostic and diagnostic prediction models has continued to expand across several medical and surgical disciplines, including orthopaedic surgery.^1–3^ Literature concerning patient populations that have undergone total shoulder arthroplasties (TSA) have also been of interest as it pertains to applying this statistical methodology,^4–10^ though this body of literature has evolved more slowly. However, rapidly growing interest in developing prognostic and diagnostic prediction models because of “hype” can lead to a substantial increase in the number of available models that fail to be implemented into clinical practice due to incomplete reporting, methodological limitations, and model bias.

A previous systematic review of AI models developed on total joint arthroplasty (TJA) datasets reported that AI models were broadly utilized in four domains (clinical outcomes and resource utilization, imaging detection, patient movement and activity surveillance, and language interpretation).^11^ However, despite the several promising applications of these prediction models, the methodological quality and reporting transparency of most studies were inadequate and failed to pursue external validation, which is imperative as healthcare institutions and device manufacturers begin to adopt such technology into clinical workflows and real-world patient care.^11^ A common lack of implementation into clinical practice and subsequently patient benefit is incomplete reporting and failure to follow such methodological recommendations, which leads to models developed on inappropriately small sample sizes, miscalibration, and failure to be externally validated. Therefore, it is essential to critically appraise studies concerning TSA given the potential clinical applicability that such algorithms will impose on everyday clinical practice and potential for methodological misconduct.

A contemporary synthesis of the efficacy and methodological conduct of clinical prediction models utilizing machine learning methods in TSA is lacking, which is critical for determining whether this evolving compilation of prediction models is methodologically valid and clinically useful or contribute to wasted research efforts. Given this importance, the primary aim of the current study was to provide a comprehensive synthesis of the current applications, performance, and methodological conduct of contemporary AI prediction models for TSA outcomes. It was hypothesized that studies evaluating AI models in the context of TSA would demonstrate good to excellent performance in clinical prediction tasks but demonstrate concerning methodological conduct and possess a high risk of bias.

## Methods

### Article identification

Four online databases (PUBMED, MEDLINE, EMBASE, and Cochrane Central Register of Controlled Trials) were queried for literature that developed clinical prognostic and diagnostic prediction models utilizing machine learning methodology from data inception to November 2, 2025. The search terms included, “artificial intelligence”, “machine learning”, “shoulder arthroplasty”, and similar phrases (**Appendix I**). Inclusion criteria were: (1) articles available in English; (2) articles presenting original data; and (3) articles that implicated machine learning methodology for clinical prediction model development. The exclusion criteria were: (1) biomechanical articles, (2) review articles, (3) case reports, (4) technical notes, (5) editorial notes, and (6) abstracts.

### Article selection process

A systematic screening approach in accordance with the 2020 Preferred Reporting Items for Systematic Reviews and Meta-Analyses (PRISMA) statement was conducted by two independent reviewers (_____) from title to full-text screening stages.^12^ Discrepancies at the title/abstract screening stage were resolved by automatic inclusion. Discrepancies at the full-text screening stage were resolved by input from a third reviewer (___). The references from the included studies were also screened using the same approach to ensure that relevant articles were not missed.

### Methodological conduct assessment

The TRIPOD (Transparent Reporting of a Multivariable Prediction Model for Individual Prognosis or Diagnosis) guidelines and the Guidelines for Developing and Reporting Machine Learning Models in Biomedical Research represent a consensus list of 22 items which best practices for reporting and designing machine learning prediction studies.^13,14^ These guidelines are used to assess studies that focus on developing, evaluating, or both developing and evaluating diagnostic or prognostic prediction models.^15^ These guidelines were chosen as they represent a widely accepted benchmark for appropriate prediction model development and have been utilized extensively among other reviews concerning methodological conduct of prediction models.^16–19^ A modified version which only contains key items of the TRIPOD guidelines were used to evaluate the quality of included studies in accordance with prior literature.^11^ The risk of bias and applicability of predictive models were evaluated using the Prediction model Risk of Bias Assessment Tool (PROBAST), which is also commonly utilized in reviews concerning methodological conduct.^20,21^ PROBAST assesses four domains via 20 signaling questions: participants, predictors, outcomes, and analysis.^20^ The overall risk of bias and applicability of predictive models are rated as “low”, “high” or “unclear” concern according to the PROBAST checklist.^20,21^

### Data extraction and statistical analysis

Two reviewers independently abstracted data of interest from included studies into a spreadsheet Microsoft Excel (Version 2016; Microsoft, Redmon, Washington), designed *a priori*. Demographic data included author, year of publication, sample size, location, and patient demographics. Data pertaining to primary and secondary outcome of interest, AI model development and testing, model performance, model validation, comparison with conventional statistical methods and whether studies were externally validated or cautioned that there was requirement of external validation, was noted.

To assess model performance across included studies, metrics such as discrimination, calibration, Brier score and decision-curve analysis were recorded when possible. Discrimination, represented through the area under the receiver operating curve (AUC), ranges from 0.5 to 1.^22^ A model with an AUC of 1.0 was considered a perfect discriminator, 0.90–0.99 was considered excellent, 0.80–0.89 was good, 0.70–0.79 was fair, and 0.51–0.69 was considered poor.^23^ Calibration slope describes the degree to which model predictions match the observed outcomes, whereas calibration intercept quantifies by how much the predictions differ from the outcomes.^24,25^ A combination of values for slope and intercept represents different levels of calibration: a slope of 1 and intercept of 0 represent perfect calibration. Calibration slopes that are >1 or <1 indicate that predictions are too extreme and too moderate, respectively, while negative and positive calibration intercepts indicate overestimation and underestimation of predictions, respectively.^24,25^ Brier scores, ranging from 0 to 1, combines discrimination and calibration to provide estimates on the accuracy of the prediction models, where a score 0 represents perfect accuracy.^26^

Due to the high statistical and methodological heterogeneity amongst included studies, a meta-analysis could not be performed. Thus, the data is presented descriptively. Descriptive statistics including counts, proportions, means, medians, ranges, and measures of variance (e.g., standard deviations [SD], 95% confidence intervals [CI]) are presented where applicable as a range of all values reported within the

## Results

### Patient and study characteristics

**Figure 1.**
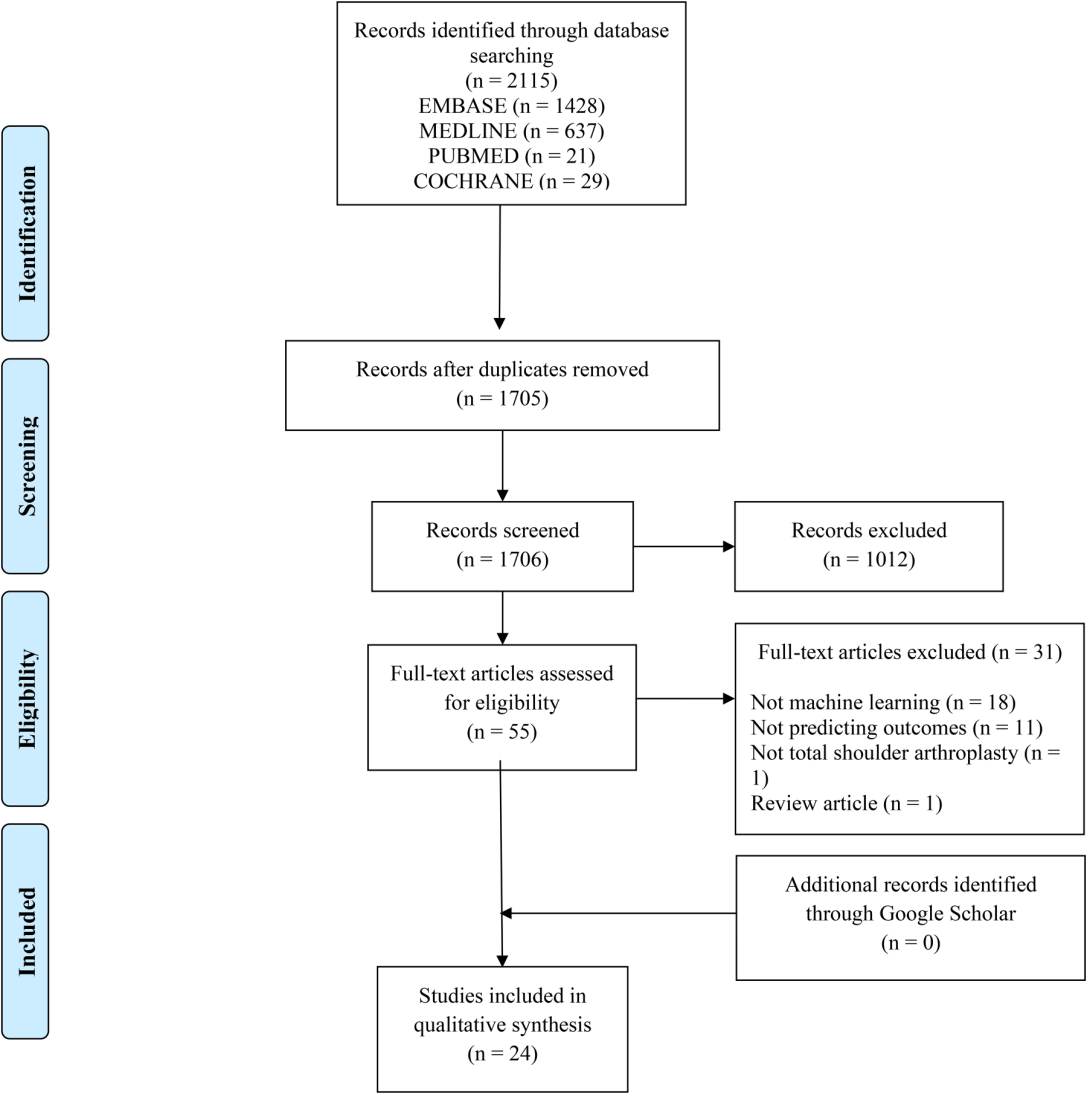
Preferred reporting items for systematic reviews and meta-analyses (PRISMA) flow diagram demonstrating the systematic review of the literature for the use of machine learning algorithms to predict outcomes, complications, and resource utilization after total shoulder arthroplasty.

The initial search yielded, 2115 studies, of which a total of 24 were included in the final analysis (Figure 1). The studies included 497,365 patients (35.6% female) with a mean age of 69.6 ± 0.9 years and mean BMI of 30.7 ± 1.5 kg/m^2^. The median sample size was 5774 (range: 60 to 178,003). Patients were treated with either rTSA (31.0%; n = 154,374), aTSA (29.1%; n = 144,920) or hemiarthroplasty (2.8%; n = 13,732; three studies did not specify the proportion between aTSA and rTSA (37.1%; 184,339)^27–29^ (**Table 1**). All studies were published in 2019 or later, with the majority (29.2%; n = 7) being published in 2021.^6–10^^,30–35^ Eleven (45.8%) studies included combined populations of patients that received either aTSA or rTSA^5–10^^,27–29,35,36^; seven (29.2%) included aTSA patients alone^4,30,^^32–34^^,37,38^; five (20.8%) rTSA patients alone^31,^^39–42^; and one (4.2%) included rTSA, aTSA and hemiarthroplasty patients.^43^ Datasets consisted of national registries (45.8%, n = 11),^4,^^27–30^^,32,34,36,37,40,43^ multi-center institutional collaborations (25.0%; n = 6),^5–7^^,10,35,41^ single institutions (20.8%; n = 5),^8,9,38,39,42^ and statewide registries (12.5%; n = 2).^31,33^ Studies were completed in the United States (n = 23; 95.8%) and Italy (n = 1; 4.2%).

**Table 1.** Study demographics.

Primary Author	Year	Field of Research	Study Population	Total Sample Size	%Female	Mean Age ± SD (range) Years	Mean BMI (range)
Gowd AK	2019	aTSA	National registry	17119	43.8	69.5 ± 9.6	31.1 ± 6.8
Biron DR	2020	aTSA	National registry	Total - 4500 Short LOS - 2122 Long LOS - 1372	38.3	Short LOS - 67.8 Long LOS - 72.8	Short LOS - 31.2 Long LOS - 31.7
Kumar V	2020	aTSA and rTSA	Multicentre	Total - 4782 aTSA – 1895 rTSA - 2887	60.1	Total - 70 ± 9 aTSA - 66 ± 9 rTSA - 72 ± 8	NR
Karnuta JM	2020	aTSA, rTSA and HSA	National registry	Total - 90792 Chronic/degenerative- 73,162 Acute/traumatic −17,630	59.2	Total - 69.0 ± 10.9 Chronic/degenerative - 68.4 ± 10.7 Acute/traumatic - 71.4 ± 11.5	NR
Kumar V	2021	aTSA and rTSA	Multicentre	Total - 5774 aTSA – 2153 rTSA - 3621	59.9	aTSA - 66.1 ± 9.2 rTSA - 72.5 ± 7.8	aTSA - 29.9 ± 6.3 rTSA - 28.7 ± 6.0
Kumar V	2021	aTSA and rTSA	Multicentre	Total - 2790 aTSA - 1141 rTSA - 1649	NR	NR	NR
Arvind V	2021	aTSA	National registry	9043	43.6	69.4 ± 0.4	NR
Polce EM	2021	aTSA and rTSA	Single centre	Total – 413 aTSA - 225 rTSA - 118	41.4	66.0 (60.0–72.0)^a^	28.6 (25.8–32.5)^a^
Devana SK	2021	rTSA	Statewide registry	2799	48.9	69 ± 12	NR
Lopez CD	2021	aTSA	National registry	Total - 21544 Home discharge - 11,397 Nonhome discharge - 10,147	55.3	Total - 69.1 ± 9.5 Home discharge - 68.4 Nonhome discharge - 75.4	Home discharge - 31.1 Nonhome discharge - 31.5
McLendon PB	2021	aTSA and rTSA	Single centre	Total - 472 aTSA - 431 rTSA - 41	43.6	68 (28–89)	NR
Devana SK	2022	aTSA	Statewide registry	10302	54.1	71 ± 12	NR
Kumar V	2022	aTSA and rTSA	Multicentre	Total - 6468 aTSA - 2270 rTSA - 4198	60.3	aTSA: 66.2 ± 9.1 rTSA: 72.6 ± 7.9	aTSA - 30.0 ± 6.4 rTSA - 28.7 ± 6.0
Lopez CD	2022	aTSA	National registry	21544^b^	55.3^b^	69.1 ± 9.5^b^	Home discharge - 31.1^b^ Nonhome discharge - 31.5^b^
Kumar V	2022	aTSA	Multicentre	Total - 6468^b^ aTSA - 2170 rTSA - 4198	60.3^b^	aTSA - 66.2 ± 9.1^b^ rTSA - 72.6 ± 7.9^b^	NR
Gowd AK	2022	aTSA	National registry	Total - 49354 aTSA - 16993 rTSA - 32361	58.0	70.2 ± 9.1	NR
Oeding JF	2023	aTSA and rTSA	National Registry	74697	70.0	62.5	NR
Schneller T	2024	rTSA	Multicentre	1707	NR	NR	NR
Franceschetti E	2024	rTSA	Single Centre	105	71.4	69.4 ± 7.6	NR
Kim A	2024	aTSA and rTSA^c^	National Registry	525	NR	NR	NR
Miltenberg B	2024	aTSA and rTSA^c^	National Registry	5811	54.9	NR	NR
Marigi EM	2025	rTSA	Single Centre	3837	54.8	71.7 ± 9.3 (20–97)	30.5 ± 6.8
Parmigiani O	2025	aTSA	Single Centre	Total – 60 aTSA – 23 rTSA – 37	48.3	69.3 ± 7.5 (53–84)	27.5 ± 3.9 (20–36)
Powell CM	2025	aTSA and rTSA^c^	National Registry	178,003	NR	NR	NR

BMI – Body Mass Index

* The TRIPOD (Transparent Reporting of a Multivariable Prediction Model for Individual Prognosis or Diagnosis) guidelines and the Guidelines for Developing and Reporting Machine Learning Models in Biomedical Research

aTSA – Anatomic Total Shoulder Arthroplasty

rTSA – Reverse Total Shoulder Arthroplasty

LOS – Length of Stay

a – Median value, demographic data not used in calculations

NR – Not Reported

b - Duplicate population, demographic data not used in calculations

c – Distribution of aTSA and rTSA was not specified in the study

### Methodological conduct assessment using TRIPOD guidelines

TRIPOD grading for each individual study is given in **Appendix II**. The mean number of criteria met across included studies was 11.6 (range: 9 to 15). Most studies (n = 19 studies; 82.3%) met greater than half of the criteria in the checklist; however, only 6 (26.1%) met greater than two-thirds of the criteria.^27,28,37,40,42,43^ Descriptions for how missing data were handled (criterion 9) were reported across 19 studies (79.2%).^4–10^^,27–29,32,34,35,37–42^ Blinding for assessing the predictors (criterion 7b) was often omitted and performed in only one (6.3%) study.^36^ Only 14 (58.3%) studies adequately described how predictors were handled during analyses (criterion 10a).^7,8,^^27–29^^,32,34,35,37–42^ Describing the flow of participants (criterion 13a) and including the number of participants with missing data (criterion 13b) was poorly reported and included in only 10 (41.7%)^28,29,37,38,^^40–43^ and eleven studies (45.8%),^9,^^27–29^^,37–43^ respectively. Presentation of the full model to allow for individual predictions (criterion 15a) was variably reported and included in only three (18.8%) studies.^8,9,31^ None of the studies explained how to use the prediction model (criterion 15b). The need and caution for external validation using independent data sets were reported in just over half of the studies (n = 15 studies; 62.5%).^6,^^8–10^^,29,31–33,35–39,41,42^

### Methodological conduct assessment using PROBAST

**Figure 2.**
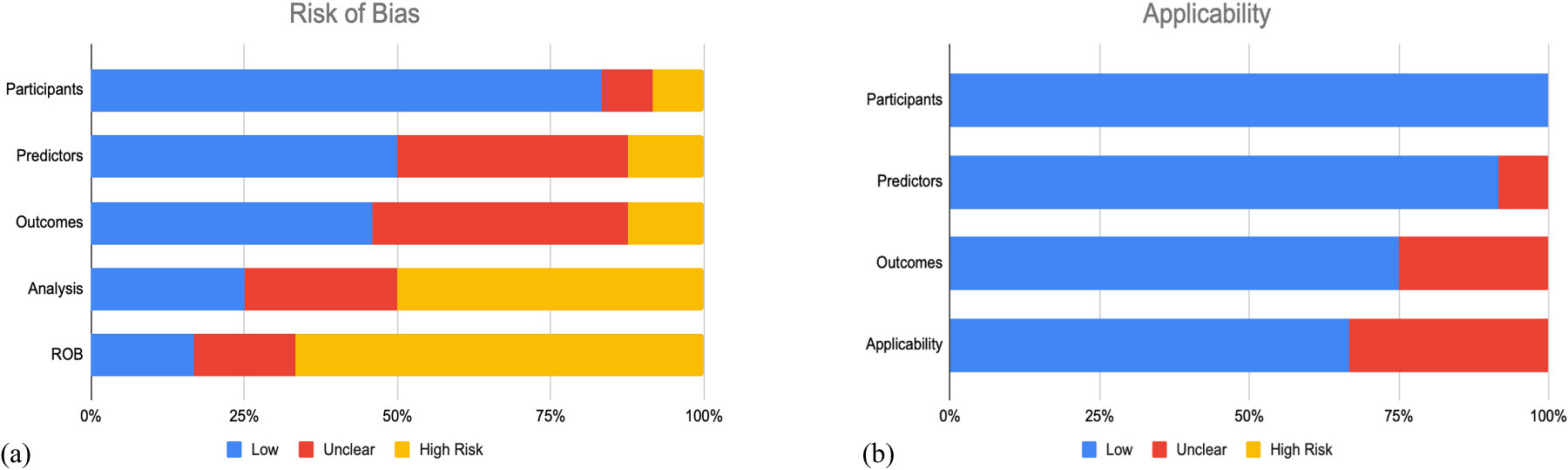
Summary of prediction model risk of bias assessment tool (PROBAST) assessment for included studies. (a) The risk of bias of the included studies. (b) The applicability of the included studies.

As depicted in Figure 2, most studies (n = 16; 66.7%) demonstrated a high risk of bias,^5–8^^,10,29–32,35–38,41–43^ whereas the remaining had an unclear risk of bias (n = 4; 25%)^4,9,33,34^ and low risk of bias (n = 4; 25%).^27,28,39,40^ The elevated risk of bias was predominately from the analysis domain, in which there was a lack of complete reporting as to how continuous and categorical predictors, complexities in the data, and model overfitting and optimism in model performance were handled. Low risk of bias was identified in the participants domain in 80.3% of studies, predictors domain for only 50% of studies, and outcomes domain for less than half (45.8%) of studies. The concern of applicability for most models were low (n = 16 studies; 66.7%) since the included participants and settings; definition, timing or assessment of predictors; and the outcome definition, timing or determination of these studies match the review question (Figure 2).^4–6^^,9,10,27,28,30,32,33,35,37–40,42,43^ The complete PROBAST assessment for each individual study is given in **Appendix III.**

### Efficacy and applications: clinical outcomes

Clinical outcomes investigated among identified studies (n = 9)^5–10^^,35,41,42^ included patient reported outcomes measures (PROMs) (n = 5 studies; 55.6%),^5–8^^,35^ range of motion (n = 2 study; 22.2%),^10,42^ and patient satisfaction (n = 1 study; 11.1%).^9^ Six studies (66.7%) reported the AUC (median [range]: 0.80 [0.52 to 0.96]),^5,6,9,10,35,41^ six studies (66.7%) reported sensitivity (median [range]: 93% [57% to 99%]),^5,6,8,10,35,41^ five studies (55.6%) reported accuracy (median [range]: 88.5% [63% to 97%]),^5,6,10,35,41^ and five studies (55.6%) reported positive predictive value (PPV) (median [range]: 87.3% [55% to 97%]).^5,6,10,35,41^ Calibration slope (2.32) and intercept (0.20) as well as Brier Score (0.11) was only reported in one study (11.1%), in which support vector machine (SVM) was the best performing AI model.^9^ The best performing model among six studies that compared multiple AI models was Wide and Deep (n = 3 studies; 75%),^5,10,35^ SVM/SVR (n = 2 studies; 22.2%),^9,42^ and ANN (n = 1 study, 11.1%).^41^ Outcomes were primarily continuous necessitating regression-based models (n = 8 studies; 88.9%)^5–8^^,10,35,41,42^ as opposed to categorical requiring classification-based models (n = 1 study; 11.%) (Table 2).^9^ The median number of features used during model training was 34 (range: 13 to 291), with all studies (n = 9) using demographic based features. ^5–10,35,41,42^With regards to internal validation, two studies (22.2%) used three to five iterations of cross validation (CV) on a training set^9,42^; while one study (11.1%) did not use any validation method as all data sets were used to train the AI models.^8^ The remaining six studies (66.7%) developed the AI algorithms by splitting the data into training and validation sets.^5–7^^,10,35,41^ Out of the studies (n = 4; 44.4%) that compared AI to traditional regression, all studies reported that AI models demonstrated superior performance (Table 3).^5,10,35,42^

### Efficacy and applications: resource utilization

Resource utilization measures evaluated across a total of 8 studies,^4,28,29,32,34,36,37,43^ included length of stay (n = 4 studies; 50%),^4,29,37,43^ discharge disposition/nonhome discharge (n = 3 studies; 37.5%),^28,32,43^ cost of care (n = 2 studies; 25.0%)^36,43^ and operative time (n = 1 study; 12.5%).^34^ All studies reported AUC (median [range]: 0.77 [0.59 to 0.91],^4,28,29,32,34,36,37,43^ five studies (62.5%) reported accuracy (median [range]: 89.9% [75.2% to 95.5%])^4,29,32,34,43^ and two studies (25.0%) reported positive predictive value (32–61.4%).^4^ No studies reported model calibration, Brier score, or decision curve analysis (Table 4). The median number of features used during model training was 21 (range: 12 to 52), with all studies using demographic input features (n = 8 studies).^4,28,29,32,34,36,37,43^ AI algorithms were developed by splitting data into training and test sets for all studies (n = 8 studies; 100%).^4,28,29,32,34,36,37,43^ Out of the studies (n = 5; 62.5%) that compared AI to traditional regression, all reported that AI models demonstrated superior performance (Table 3).^4,28,29,34,36^

**Table 2.** AI performance in tasks pertaining to clinical outcomes.

Primary Author, Year	Field of Research	AI Model Used	MAE	PPV (%)	Sensitivity (%)	Accuracy (%)	AUC	F1 Score	Performance vs Standard Regression	Caution Need for External Validation
Kumar V, 2020	aTSA and rTSA	Wide and Deep (XGBoost, DL)	ASES: 10.2 (10.1–11.3) UCLA: 2.75 (2.5–3.4) Constant: 7.5 (7.3–7.9) VAS Pain: 1.2 (1.2–1.4) Abduction: 18.7 (18.0–21.3) Forward Elevation: 15.25 (15.1–17.1) External Rotation: 11.6 (10.0–11.9)	MCID: 93 (91–97) SCB: 86.5 (81–90)	MCID: 98 (96–99) SCB: 92.5 (91–99)	MCID: 93.5 (90–96) SCB: 85.5 (82–90)	MCID: 0.895 (0.84–0.94) SCB: 0.855 (0.78–0.88)	-	Outperformed	No
Kumar V, 2021	aTSA and rTSA	XGBoost	Weighted MAE: Full: 11.7 (1.3–20.4) Abbreviated: 12.0 (1.4–21.8) Abbreviated + Implant: 12.0 (1.3–21.7)	MCID ASES: 95% Constant: 96% VAS: 92%	MCID ASES: 99% Constant: 99% VAS: 98%	MCID ASES: 95% Constant: 97% VAS: 93%	ASES: 0.90 Constant: 0.95 VAS: 0.87	-	NA	No
Polce EM, 2021	aTSA and rTSA	SVM	-	-	-	-	AUC: 0.80 (0.64–0.90)	Calibration intercept: 0.20 (-0.40,0.81) Calibration slope: 2.32 (0.92–3.71) Brier score: 0.11 (0.06–0.15)	NA	Yes
Kumar V, 2022	aTSA and rTSA	XGBoost (Full, Minimal)	Normalized MAE: Full: 1.09 (0.98–1.14) Minimal: 1.09 (0.98–1.14)	MCID: 86% SCB: 90%	MCID: 92% SCB: 83%	MCID: 85% SCB: 81%	MCID: 0.82 SCB: 0.81	MCID: 89% SCB: 85%	NA	Yes
McLendon PB, 2021	aTSA and rTSA	Tree, SVM, KNN	-	-	Sensitivity (2-year follow-up): Tree: 0.84–0.95 SVM: 0.69–0.92 KNN: 0.59–0.72	Sensitivity (>2-year follow-up): Tree: 0.91–0.98 SVN: 0.57–0.96 KNN: 0.60–0.86	-	-	NA	Yes
Kumar V, 2021	aTSA and rTSA	XGBoost	UCLA F-score: -1563 ± 1095 Constant F-score: -879 ± 1140 ASES F-score: 321 ± 496	-	-	-	-	Reciprocal fusion rank: UCLA: -0.032 ± 0.05 Constant: -0.027 ± 0.005 ASES: -0.028 ± 0.001	NA	No
Kumar V, 2022	aTSA and rTSA	XGBoost	Normalized MAE: ASES: 10.68 Constant: 8.25 SAS: 7.56	MCID: SAS: 97% Constant: 97% ASES: 95%	MCID: SAS: 99% Constant: 99% ASES: 99%	MCID: SAS: 97% Constant: 97% ASES: 95%	MCID: SAS: 0.96 Constant: 0.95 ASES: 0.90	MCID: SAS: 98% Constant: 98% ASES: 97%	NA	Yes
Franceschetti E, 2024	rTSA	LR, SVR	SVR: 11.6 LR 12.99	-	-	-	-	-	Outperformed	Yes
Schneller T, 2024	rTSA	ANN, RF, XGBoost	-	-	Negative class (no pain)*: ANN: 0.67 RF: 0.84 XGBoost: 0.88 Positive class (pain)*: ANN: 0.57 RF: 0.34 XGBoost: 0.29	ANN: 63% RF: 63% XGBoost: 63%	ANN: 0.65	Negative class (no pain)*: ANN: 0.68 RF: 0.72 XGBoost: 0.74 Positive class (pain)*: ANN: 0.56 RF: 0.43 XGBoost: 0.40	NA	Yes

aTSA – Anatomic Total Shoulder Arthroplasty; rTSA – Reverse Total Shoulder Arthroplasty; MCID – Minimum Clinically Important Difference; SCB – Substantial Clinical Benefit; ASES – American Shoulder and Elbow Surgeons Score; UCLA – University of California, Los Angeles Shoulder Score; Constant – Constant Shoulder Score; Global Shoulder Function – A composite score evaluating overall shoulder function; VAS – Visual Analog Scale (Pain Assessment); SAS – Shoulder Arthroplasty Smart Score; ROM – Range of Motion; GHOA – Glenohumeral Osteoarthritis; MAE – Mean Absolute Error; PPV – Positive Predictive Value; Sensitivity – Measure of true positive rate; Accuracy – Overall correctness of the model; AUC – Area Under the Receiver Operating Characteristic Curve; F1-score – Harmonic mean of precision and recall; Reciprocal Fusion Rank Score – A ranking metric used for fusion models; Brier Score – A measure of the accuracy of probabilistic predictions; Calibration Intercept & Slope – Measures of how well predicted probabilities align with observed outcomes; C-statistic – Concordance statistic, similar to AUC; XGBoost – Extreme Gradient Boosting; Deep Learning – A subset of machine learning using neural networks; Stochastic GB – Stochastic Gradient Boosting; RF – Random Forest; SVM – Support Vector Machine; NN – Neural Network; Elastic-Net Penalized LR – Elastic-Net Regularized Logistic Regression; Feature Set (Full, Minimal, Abbreviated, Implant/X-Ray Data) – Different AI input feature groups; Complete Case Analysis – Statistical method where only complete data cases are analyzed; Multiple Imputation – A statistical method for handling missing data; LR – Linear Regression; SVR – Support Vector Regression; * - Negative class (“no pain”): Numeric Rating Scale 0–2, Positive Class (“pain”): Numeric Rating Scale 3+

**Table 3.** Summary of AI models across included studies.

Primary Author, Year	Field	Outcome(s)	AI Models	Inputs (No.)	Validation	vs Regression	Ext. Validation
Gowd AK, 2019	aTSA	Extended LOS, postop complications	KNN, RF, GB, others	Demo, med, lab (22)	Split train/test	Similar or Outperformed	No
Biron DR, 2020	aTSA	Extended LOS	RF	Demo, med (12)	Split train/test	NA	Yes
Karnuta JM, 2020	aTSA, rTSA, HA	LOS, discharge, charges	NN	Demo, med	Split train/test/valid	NA	Yes
Kumar V, 2020	aTSA, rTSA	Postop outcomes, MCID, SCB	XGBoost, DL	Demo, radio, med (291)	Split train/test	Outperformed	No
Arvind V, 2021	aTSA	30-day readmissions	SVM, RF, AB, NN	Demo, med, lab (32)	Split train/test	Outperformed	Yes
Devana SK, 2021	rTSA	Complications, 30-day readmission	RF, AB, GB, XGBoost	Demo, med, hosp (64)	5-fold CV	Outperformed	No
Kumar V, 2021	aTSA, rTSA	Postop outcomes	XGBoost	Demo, radio, med (291)	Split train/test	NA	No
Kumar V, 2021	aTSA, rTSA	ASES, Const, UCLA, SST, SPADI	XGBoost	Demo, radio, med (291)	Split train/test	NA	No
Lopez CD, 2021	aTSA	Nonhome discharge, 30-day complications	Boosted DT, NN	Demo, med, surg (21)	Split train/test	NA	Yes
McLendon PB, 2021	aTSA, rTSA	2-yr max improvement	Tree, SVM, KNN	Demo, radio (13)	NA	NA	Yes
Polce EM, 2021	aTSA, rTSA	2-yr satisfaction	SVM, RF, NN, others	Demo, med (16)	NS	NA	Yes
Devana SK, 2022	aTSA	Complications or readmission	XGBoost, GB, RF	Demo, med, hosp (64)	5-fold CV	Outperformed	Yes
Gowd AK, 2022	aTSA, rTSA	Increased cost#, postop readmission	KNN, RF, GB, others	Demo, med, hosp (12)	Split train/test	Similar or Outperformed	Yes
Kumar V, 2022	aTSA, rTSA	Postop internal rotation	XGBoost, DL	Demo, med, surg (291)	Split train/test	NA	Yes
Kumar V, 2022	aTSA, rTSA	SAS, ASES, Const scores	XGBoost, DL	Demo, radio, med (291)	Split train/test	NA	Yes
Lopez CD, 2022	aTSA	Prolonged op time*, postop complications	Boosted DT, NN	Demo, med, surg (21)	Split train/test	Similar or Outperformed	No
Oeding JF, 2023	rTSA	Dislocation within 90 days	XGBoost, SVM, RF, CNB, L2PLR	Demo, Med, Surg, Outcome (20)	Split train/test, 10-fold cross validation	Similar	No
Schneller T, 2024	rTSA	Postoperative pain	ANN, RF, XGBoost	Demo, Med, Clinical, PROMs (34)	Split train/test	NA	Yes
Franceschetti E, 2024	rTSA	Postoperative anterior elevation	LR, SVR	Demo, Clinical, Radio, Surg (28)	Split train/test	NA	Yes
Kim A, 2024	aTSA	Reoperation	Ensemble of RF, LR, SVM, AB	Demo, Med, Surg, Lab (37)	Split train/test	NA	No
Miltenberg B, 2024	aTSA	Overnight hospital stay	RF, ANN, GB, Naïve Bayes, SVM	Demo, Periop, Postop (14)	Split train/test	Outperformed	No
Marigi EM, 2025	rTSA	Complications	XGBoost	Demo, Surg (rTSA design) (34)	5-fold CV	NA	Yes
Parmigiani O, 2025	aTSA	Complications	LR, GB, SVM, Multilayer perceptron classifier	Demo, Clinical, Radio, Surg (23)	Split train/test	Outperformed	Yes
Powell CM, 2025	aTSA and rTSA	Complication within 180 days Extended length of stay (LOS>2 days)	Weighted LR, RF, GB, ANN	Demo, Med, Hosp (52)	Split train/test	Similar or Outperformed	Yes

aTSA – Anatomic Total Shoulder Arthroplasty; rTSA – Reverse Total Shoulder Arthroplasty; HA – Hemiarthroplasty; LOS – Length of Stay; Op – Operative; Hosp – Hospital; Demo – Demographic; Radio – Radiographic; Med – Medical; Lab – Laboratory; Surg – Surgical; Valid – Validation; DL – Deep Learning; KNN – K-nearest Neighbor; RF – Random Forest; GB – Gradient Boosting; NN – Neural Network; DT – Decision Tree; Boosted DT – Boosted Decision Tree; SVM – Support Vector Machine; AB – Adaptive Boosting; XGBoost – Extreme Gradient Boosting; MCID – Minimum Clinically Important Difference; SCB – Substantial Clinical Benefit; ASES – American Shoulder and Elbow Surgeons; Const – Constant; UCLA – University of Los Angeles California; SST – Simple Shoulder Test; SPADI – Shoulder Pain and Disability Index; SAS – Shoulder Arthroplasty Smart; NS – Not Specified; CV – Cross Validation; NA – Not Applicable; * – Prolonged operative time: >150 min; # – Increased cost: $32,883 (1 SD above average); SVR – Support Vector Regression; CNB – Complement Naïve Bayes; L2PLR – L2 Penalized Logistic Regression

### Efficacy and applications: adverse events

Adverse events were reported across 12 studies,^4,27,^^29–34^^,36,38–40^ and included all-cause adverse events (complications, reoperations) (n = 10 studies; 83.3%)^4,27,29,^^31–34^^,38–40^ and unplanned readmission (n = 4 studies; 44.4%).^30,31,33,36^ 12 studies (100%) reported AUC (median [range]: 0.71 [0.49 to 0.92]),^4,27,^^29–34^^,36,38–40^ seven studies (58.3%) reported accuracy (median [range]: 95.3% [20.8% to 99.6%]),^4,27,29,32,34,38,39^ four studies (33.3%) reported Brier score (median [range]: 0.150 [0.037 to 0.052])^31,33,38,40^ and PPV (range: 52.2 to 62.5) (Table 5).^4,29,36^ The median number of features used during model training was 25 (range: 12 to 64), with all studies using demographic and medical data as input features (n = 12 studies).^4,27,^^29–34^^,36,38–40^ AI algorithms were developed by splitting data into training and test sets for all studies (n = 12 studies).^4,27,29,30,32,34,36,^^38–40^ In terms of internal validation methods, CV (n = 4 studies) was used.^27,31,33,40^ Out of the studies (n = 9; 75.0%) which compared AI to traditional regression, all reported that AI models demonstrated superior performance. (Table 3).^4,^^29–31^^,33,34,36,38,40^

**Table 4.** AI performance in tasks pertaining to resource utilization.

Primary Author, Year	Field of Research	Outcome(s) of Interest	AI Models Used †	Accuracy	AUC	Other Metrics	Performance vs Standard Regression	Caution Need for External Validation
Gowd AK, 2019	aTSA	Extended LOS	K-nearest neighbor, RF, Naive-Bayes, Decision Tree, GB	82.1% (RF/GB)	0.70 (GB)	PPV: 61.4 (RF)	Similar or Outperform	No
Biron DR, 2020	aTSA	Extended LOS	RF	NR	0.77	NR	NA	Yes
Karnuta JM, 2020	aTSA, rTSA, HA	LOS, discharge disposition, and inpatient charges	NN	91.80%	0.89	NR	NA	Yes
Lopez CD, 2021	aTSA	Nonhome discharge	Boosted decision trees, NN	90.3% (Boosted decision tree)	0.851 (NN)	NR	NA	Yes
Lopez CD, 2022	aTSA	Prolonged operative time*	Boosted decision trees, NN	85.6% (Boosted decision tree)	0.906 (NN)	NR	Similar or Outperform	No
Gowd AK, 2022	aTSA, rTSA	Increased total cost of care#	K-nearest neighbor, RF, Naive-Bayes, Decision Tree, GB	NR	0.87 (GB)	NA	Similar or Outperform	Yes
Miltenberg B, 2024	aTSA	Overnight Stay	RF, ANN, GB, Naïve Bayes, SVM	NR	ANN 0.811, RF 0.755, GB 0.727, Naïve Bayes 0.719, SVM 0.623, Multivariable LR 0.703			
Powell CM, 2025	aTSA and rTSA	Extended length of stay (LOS>2 days)	Weighted LR, RF, GB, ANN	LR 0.72, RF 0.85, GBC 0.76, ANN 0.79	LR 0.76, RF 0.78, GBC 0.80, ANN 0.82	Precision (PPV) – LR 0.32, RF 0.56, GBC 0.36, ANN 0.43 Recall (Sensitivity) – LR 0.64, RF 0.32, GBC 0.67, ANN 0.67 F1 Score – LR 0.42, RF 0.41, GBC 0.47, ANN 0.52	Similar or Outperform	Yes

†For studies directly comparing multiple machine learning models, the best performing algorithm's metrics are provided

aTSA – Anatomic Total Shoulder Arthroplasty; LOS – Length of Stay; RF – Random Forest; GB – Gradient Boosting; AUC – Area Under the Receiver Operating Characteristic Curve; PPV – Positive Predictive Value; NA – Not Applicable; rTSA – Reverse Total Shoulder Arthroplasty; HA – Hemiarthroplasty; NN – Neural Network; * – Defined > 150 min or approximately one standard deviation above the mean operative time; # – $32,883, which represents 1 standard deviation greater than the average cost.

**Table 5.** AI performance in tasks pertaining to complications.

Primary Author, Year	Field of Research	Outcome(s) of Interest	AI Models Used †	Accuracy	AUC	Other Metrics	Performance vs Standard Regression	Caution Need for External Validation
Gowd AK, 2019	aTSA	Postoperative complication	K-nearest neighbor, RF, Naive-Bayes, Decision Tree, GB	95.3% (GB)	0.71 (GB)	PPV: 62.5 (GB); Accuracy: 99.6 (KNN/RF)	Similar or Outperform	No
Arvind V, 2021	aTSA	30-day unplanned readmissions	SVM, RF, AB, NN	0.74 (RF)	NR	F1 score: 0.18 (RF); Sensitivity: 0.91 (NN)	Outperform	Yes
Devana SK, 2021	rTSA	Postoperative complication and 30-day readmission	XGBoost, GB, AB, RF	NR	0.681 ± 0.064 (XGBoost)	AUPRC: 0.129 ± 0.049 (XGBoost); Brier Score: 0.037 ± 0.002 (XGBoost)	Outperform	No
Lopez CD, 2021	aTSA	30-day postoperative complications	Boosted decision trees, NN	95.5% (Boosted decision tree)	0.795 (Boosted decision tree)	NR	NA	Yes
Devana SK, 2022	aTSA	Any major complication or readmission	XGBoost	NR	0.689 ± 0.026 (XGBoost)	AUPRC: 0.214 ± 0.049 (GB); Brier Score: 0.051 ± 0.002 (XGBoost/GB)	Outperform	Yes
Lopez CD, 2022	aTSA	Postoperative complication	Boosted decision trees, NN	95.5% (Boosted decision tree/NN)	0.795 (Boosted decision tree)	NR	Similar or Outperform	No
Gowd AK, 2022	aTSA, rTSA	Postoperative readmission	RF, GB	NR	0.66 (RF/GB)	NR	Outperform	Yes
Oeding JK, 2023	rTSA	Dislocation within 90 days	XGBoost, SVM, RF, CNB, L2PLR	NR	XGBoost: 0.71 (95% CI 0.70–0.72) SVM: 0.52 (95% CI 0.51–0.54) RF: 0.64 (95% CI 0.63–0.65) CNB: 0.68 (95% CI 0.67–0.69) L2PLR: 0.70 (95% CI 0.70–0.72)	F2 score XGBoost: 0.07 (95% CI 0.07–0.07) SVM: 0.05 (95% CI 0.05–0.05) RF: 0.06 (95% CI 0.06–0.06) CNB: 0.07 (95% CI 0.06–0.07) L2PLR: 0.07 (95% CI 0.07–0.07) Recall (Sensitivity) XGBoost: 0.84 (95% CI 0.82–0.85) SVM: 0.87 (95% CI 0.85–0.88) RF: 0.82 (95% CI 0.80–0.84) CNB: 0.80 (95% CI 0.78–0.81) L2PLR: 0.83 (95% CI 0.81–0.85) Brier score XGBoost: 0.21 (95% CI 0.20–0.21) SVM: 0.52 (95% CI 0.51–0.53) RF: 0.24 (95% CI 0.23–0.25) CNB: 0.23 (95% CI 0.23–0.24) L2PLR: 0.22 (95% CI 0.21–0.22)	Similar or Outperform	No
Kim A, 2024	aTSA	Postoperative Reoperation	Ensemble of RF, LR, SVM, AB	0.852	0.91	Weighted F1 0.85	NA	No
Marigi EM, 2025	rTSA	Postoperative complications	XGBoost	0.87 (95%CI 0.85–0.90)	0.61	NR	NA	Yes
Parmigiani O, 2025	aTSA	Postoperative complications	LR, GB, SVM, Multilayer perceptron classifier	LR 0.80, GBC 0.87, SVM 0.73, MLPC 0.67 Balanced accuracy - LR 0.88, GBC 0.92, SVM 0.71, MLPC 0.67	LR 0.833, GBC 0.916, SVM 0.861, MLPC 0.806 PR-AUC - LR 0.372, GBC 0.656, SVM 0.777, MLPC 0.754	Precision (PPV) - LR 0.50, GBC 0.60, SVM 0.40, MLPC 0.33 Recall (sensitivity) - LR 1.00, GBC 1.00, SVM 0.67, MLPC 0.67 F1 score - LR 0.67, GBC 0.75, SVM 0.50, MLPC 0.44 Brier score (Calibration) - LR 0.187, GBC 0.148, SVM 0.150, MLPC 0.326	Outperformed	Yes
Powell CM, 2025	aTSA and rTSA	Postoperative complication within 180 days	Weighted LR, RF, GB, ANN	Any Complication Within 180 Days: LR 0.65, RF 0.88, GBC 0.70, ANN 0.71 Mechanical Complication: LR 0.60, RF 0.64, GBC 0.72, ANN 0.60	Any Complication Within 180 Days LR 0.65, RF 0.67, GBC 0.71, ANN 0.69 Mechanical Complication: LR 0.62, RF 0.73, GBC 0.77, ANN 0.75	Any Complication Within 180 Days: Precision (PPV) – LR 0.18, RF 0.38, GBC 0.21, ANN 0.21 Recall (Sensitivity) – LR 0.55, RF 0.08, GBC 0.59, ANN 0.53 F1 Score – LR 0.27, RF 0.13, GBC 0.31, ANN 0.30 Mechanical Complication: Precision (PPV) – LR 0.04, RF 0.04, GBC 0.05, ANN 0.04 Recall (Sensitivity) - LR 0.58, RF 0.67, GBC 0.53, ANN 0.77 F1 Score – LR 0.07, RF 0.08, GBC 0.08, ANN 0.08	Similar or Outperform	Yes

†For studies directly comparing multiple machine learning models, the best performing algorithm's metrics are provided

aTSA – Anatomic Total Shoulder Arthroplasty; RF – Random Forest; GB – Gradient Boosting; AUC – Area Under the Receiver Operating Characteristic Curve; PPV – Positive Predictive Value; AB – Adaptive Boosting; SVM – Support Vector Machine; NN – Neural Network; NA – Not applicable; NR – Not Reported; rTSA – Reverse Total Shoulder Arthroplasty; XGBoost – Extreme Gradient Boosting; AUPRC – Area Under Precision-Recall Curve; SVM – Support Vector Machine; CNB – Complement Naïve Bayes; L2PLR – L2 Penalized Logistic Regression; PR-AUC - Area under the Precision–Recall curve;

## Discussion

The principle findings of the current study are as follows: (1) current applications of AI in the TSA literature included prediction models developed on datasets to predict clinical outcomes, adverse events, and resource utilization after TSA; (2) clinical prediction models demonstrated fair to excellent performance in predicting clinical outcomes and resource utilization, but poor performance for predicting adverse events on TSA datasets, with all studies comparing AI and traditional regression models reporting superior performance in favor of AI; (3) although most studies met greater than half of the criteria outlined in the TRIPOD guidelines, only 26.1% met greater than two-thirds of criteria, suggesting a concerning degree of incomplete and non-transparent reporting in this literature; and (4) there was also a high risk of bias in model development and reporting as assessed with the PROBAST tool, found primarily in the analysis domain. Therefore, the current review provides evidence of poor methodological conduct and unacceptable reporting quality for existing clinical prediction models developing using machine learning but identifies several areas for improvements moving forward.

Several other domains of AI have been investigated in the context of TSA including imaging detection, language interpretation and clinical decision support. In the current study that focused on clinical prediction models derived from machine learning approaches, it was identified that the current applications include prognostication of clinical outcomes, resource utilization (i.e., length of stay, operative time, cost of care) and adverse events. Given the recent introduction of AI in orthopedic surgery and rapidly evolving technologic capabilities,^1–3^ it is both plausible and likely that applications for TSA will expand over time. For example, an important and developing application of AI is patient procedural indication via risk stratification.^37^ AI models can help personalize treatment for patients by providing predictive analytics based on specific medical profiles when indicating patients for a shoulder arthroplasty procedure.^4^ Furthermore, implementation of such models may also help determine the most appropriate and cost-effective setting for a TSA to receive their care (i.e., inpatient, outpatient, or ambulatory care) based on the risk of post-operative complications and need for associated medical infrastructure to address adverse events that may be experienced.^4,37^ Therefore, future studies are necessary to confirm the efficacy and generalizability of the current TSA models in prospective settings. Furthermore, performance confirmation of current models in the setting of expanding technological capabilities concerning integration with cloud-based platforms may lead to the development of more sophisticated provider-facing tools. Such AI-based platforms may enhance and expedite clinical workflow and prognostication through automated risk stratification that incorporates clinically relevant metrics from patient imaging autonomously through leveraging generative AI and deep learning.

The performance of AI models, as predominately reported through the AUC, was good to excellent across models developed for PROMs and resource utilization prediction, whereas performance was overall fair for predicting adverse events. Furthermore, the performance of current AI models exceeded that of traditional regression in all cases, suggesting clinical utility in applying these advanced methodologies. This performance advantage may be a function of the capability of AI models for identifying nonlinear, complex relationships, which is generally limited by conventional statistical methods.^5,6^^43–48^ Indeed, conventional statistical methods, such as regression, are considered static and rely on predefined relationships, thus making it less viable for large data sets.^43^ Regardless, these comparisons are essential in such investigations in order to avoid the inappropriate repackaging of data for AI models that may not be superior to regression. With the shift towards performing TSA in the outpatient setting due to advances in multimodal pain management and the need to decrease healthcare costs,^37^^49–51^ it will become increasingly important that predictive models demonstrate high accuracy and performance to carefully select patients via risk stratification. Further studies are needed to understand the variance in performance of current AI models developed to predict adverse events. Furthermore, it is especially concerning that current investigators have omitted evaluating model calibration, Brier score, and decision-curve analyses, as these assessments are imperative to understand the clinical utility and performance of prediction models. Until these evaluations are performed and tested, current AI models for TSA should not be used to support or augment clinical decisions, as dependence on the AUC alone can be misleading as it pertains to model behavior.

There exist numerous areas of improvement for AI models concerning TSA despite the substantial increase in literature and attention this subject has garnered. A considerable proportion of studies not only failed to specify study setting and other important methodological considers, but model transparency and reporting were consistently limited. Essential methodological considerations, such as disclosing the proportion of missing data, in addition to code source and availability, was variably reported. Moreover, only six of the identified studies met greater than two-thirds the recommended reporting criteria, which is of concern.^27,28,37,40,42,43^ Furthermore, only 62.5% of studies recommended and emphasized the importance of externally validating their models. Therefore, the authors cannot recommend, and in fact must caution against, any use of the models in the current study for these reasons. To improve upon current models, several approaches should be pursued.^6,9^ First, source code and datasets should be openly available to readers such that efforts are not wasted on repackaging data in similar manners. Furthermore, this can potentially help improve performance of AI models as this allows for others to critically appraise the methods of such investigations, which may lead to the identification of areas where changes to code or data handling boost model performance. Second, data sources should be thoroughly investigated prior to model development, as factors data missingness, distribution, and how the data was collected and whether it is regularly audited may influence the quality of the data. Third, investigators must adhere to TRIPOD reporting guidelines. These guidelines were created for prognostic and diagnostic prediction models to ensure reporting transparency and will ensure that prediction models are created on a legitimate statistical foundation. This will translate into increased confidence in models and results following publication. Fourth, investigators must not recommend use of their models until fully validated, which means prospective, international, and external validation efforts. External validity is an essential component of the model development and deployment process. Lastly, the high risk of bias across majority of included studies, as assessed by the PROBAST tool is concerning. The high risk of bias across many of the studies can be attributed to the analysis domain in which studies failed to discern whether the selection of predictors was based on univariable analysis and if the complexities in the data (e.g., censoring, competing risks, etc.) were accounted for. This can instill several biases as potential interactions or confounders among predictors are not accounted for. Furthermore, the inadequate handling of complexities can lead to suboptimal model performance and generalizability. These findings highlight the dire need to develop and validate prediction models which have robust data handling strategies and adequate multivariable analysis techniques. Henceforth, future studies assessing machine learning prediction models in the context of TSA should strive to adhere to the guidelines set forth by PROBAST, particularly with the analysis domain.

## Limitations

Several limitations are important to consider when interpreting the results of the current systematic review. First, it is limited by the quality and composition of included studies. Most studies (95.8%) were conducted in the United States, making the results less generalizable to other populations. Additionally, most studies did not specify the indications of TSA and therefore the utility of AI models for certain populations (i.e., revision, proximal humeral fractures, rotator cuff tendinopathy, etc.) could not be assessed. Future studies should develop AI models for specific populations, which may reduce heterogeneity and enhance performance. Second, due to the nature of AI models, the current review was unable to determine how individual patient factors or imaging findings affected the predictive ability of models. Third, a formal quantitative meta-analysis was unable to be performed given the heterogeneity in primary outcomes of the included studies; however, the inclusion of studies applying AI to a wide variety of predictive tasks was the purpose of the current study and allows for a scoping perspective of contemporary uses of AI in TSA literature. Next, although several studies concluded that AI models were ‘superior’ to traditional regression, these claims were based almost entirely on discrimination metrics such as accuracy and AUC. Given that calibration metrics were universally absent, these findings should be interpreted with caution. Future work should incorporate calibration to meaningfully compare AI and regression models and to determine whether the added complexity of AI methods provides true clinical value. Finally, the overall generalizability of AI prediction models and the propensity for implementation into clinical practice cannot be analyzed or recommended as no studies represented an external validation phase of algorithm development.

## Conclusion

AI prediction models in TSA show poor methodology, especially in calibration, sample size, missing data, and validation, warranting cautious interpretation and clearer direction for future research.

## Supplemental Material

sj-docx-1-sel-10.1177_17585732251412368 - Supplemental material for Transparency of reporting and methodological conduct of prognostic and diagnostic clinical prediction models developed using machine learning in total shoulder arthroplasty: A systematic review and critical appraisal Supplemental material, sj-docx-1-sel-10.1177_17585732251412368 for Transparency of reporting and methodological conduct of prognostic and diagnostic clinical prediction models developed using machine learning in total shoulder arthroplasty: A systematic review and critical appraisal by Ajaykumar Shanmugaraj, Bushra Khalid, Mithilesh V Kumar, Kyle N Kunze and Ujash Sheth in Shoulder & Elbow

sj-docx-2-sel-10.1177_17585732251412368 - Supplemental material for Transparency of reporting and methodological conduct of prognostic and diagnostic clinical prediction models developed using machine learning in total shoulder arthroplasty: A systematic review and critical appraisal Supplemental material, sj-docx-2-sel-10.1177_17585732251412368 for Transparency of reporting and methodological conduct of prognostic and diagnostic clinical prediction models developed using machine learning in total shoulder arthroplasty: A systematic review and critical appraisal by Ajaykumar Shanmugaraj, Bushra Khalid, Mithilesh V Kumar, Kyle N Kunze and Ujash Sheth in Shoulder & Elbow

sj-docx-3-sel-10.1177_17585732251412368 - Supplemental material for Transparency of reporting and methodological conduct of prognostic and diagnostic clinical prediction models developed using machine learning in total shoulder arthroplasty: A systematic review and critical appraisal Supplemental material, sj-docx-3-sel-10.1177_17585732251412368 for Transparency of reporting and methodological conduct of prognostic and diagnostic clinical prediction models developed using machine learning in total shoulder arthroplasty: A systematic review and critical appraisal by Ajaykumar Shanmugaraj, Bushra Khalid, Mithilesh V Kumar, Kyle N Kunze and Ujash Sheth in Shoulder & Elbow

## Appendix Table 1. Search strategy

**Table:**

**EMBASE:** 1428 studies	**MEDLINE:** 637 studies	**PUBMED:** 21 studies	**COCHRANE:** 29 studies
**Strategy:** 1. exp Shoulder/ or exp Shoulder Joint/ or shoulder*.mp. 2. glenohumeral*.mp. 3. exp Humerus/ or humer* 4. exp Glenoid cavity/ or glenoid.mp. 5. 1 or 2 or 3 or 4 6. exp Orthopedics/ or orthopedics.mp. 7. exp Orthopedic procedures/ or orthopedic procedures.mp. 8. orthop*.mp. 9. exp Arthroplasty, Replacement/ or exp Arthroplasty/ or arthroplasty.mp. or exp Arthroplasty, Replacement. Shoulder/ 10. shoulder arthroplasty.mp. 11. total shoulder arthroplasty.mp. 12. anatomic*.mp. 13. hemiarthroplasty.mp. or exp Hemiarthroplasty/ 14. reverse total shoulder arthroplasty.mp. 15. osteoarthr*.mp. or exp Osteoarthritis/ 16. 8 and 15 17. 6 or 7 or 8 or 9 or 10 or 11 or 12 or 13 or 14 or 15 or 16 18. exp Artificial Intelligence/ or artificial intelligence.mp. 19. exp Neural Networks, Computer/ or neural network.mp. 20. exp Machine Learning/ or machine learning.mp. 21. machine intelligence.mp. 22. exp Algorithms/ or algorithms.mp. 23. exp Deep Learning/ or deep learning.mp 24. artificial neural network.mp. 25. 18 or 19 or 20 or 21 or 22 or 23 or 24 26. predict*.mp. 27. predictive value of test.mp. or exp “Predictive Value of Tests"/ 28. score.mp. 29. scores.mp. 30. scoring system*.mp. 31. observ*.mp. 32. observer variation.mp. or exp Observer Variation/ 33. detect*.mp. 34. evaluat*.mp. 35. analy*.mp. 36. assess*.mp. 37. measure*.mp. 38. area under curve.mp. or exp Area Under Curve/ 39. ROC curve.mp. or exp ROC Curve/ 40. 26 or 27 or 28 or 29 or 30 or 31 or 32 or 33 or 34 or 35 or 36 or 37 or 38 or 39 41. 5 and 17 and 25 and 40	**Strategy:** 1. exp Shoulder/ or exp Shoulder Joint/ or shoulder*.mp. 2. glenohumeral*.mp. 3. exp Humerus/ or humer* 4. exp Glenoid cavity/ or glenoid.mp. 5. 1 or 2 or 3 or 4 6. exp Orthopedics/ or orthopedics.mp. 7. exp Orthopedic procedures/ or orthopedic procedures.mp. 8. orthop*.mp. 9. exp Arthroplasty, Replacement/ or exp Arthroplasty/ or arthroplasty.mp. or exp Arthroplasty, Replacement. Shoulder/ 10. shoulder arthroplasty.mp. 11. total shoulder arthroplasty.mp. 12. anatomic*.mp. 13. hemiarthroplasty.mp. or exp Hemiarthroplasty/ 14. reverse total shoulder arthroplasty.mp. 15. osteoarthr*.mp. or exp Osteoarthritis/ 16. 8 and 15 17. 6 or 7 or 8 or 9 or 10 or 11 or 12 or 13 or 14 or 15 or 16 18. exp Artificial Intelligence/ or artificial intelligence.mp. 19. exp Neural Networks, Computer/ or neural network.mp. 20. exp Machine Learning/ or machine learning.mp. 21. machine intelligence.mp. 22. exp Algorithms/ or algorithms.mp. 23. exp Deep Learning/ or deep learning.mp 24. artificial neural network.mp. 25. 18 or 19 or 20 or 21 or 22 or 23 or 24 26. predict*.mp. 27. predictive value of test.mp. or exp “Predictive Value of Tests"/ 28. score.mp. 29. scores.mp. 30. scoring system*.mp. 31. observ*.mp. 32. observer variation.mp. or exp Observer Variation/ 33. detect*.mp. 34. evaluat*.mp. 35. analy*.mp. 36. assess*.mp. 37. measure*.mp. 38. area under curve.mp. or exp Area Under Curve/ 39. ROC curve.mp. or exp ROC Curve/ 40. 26 or 27 or 28 or 29 or 30 or 31 or 32 or 33 or 34 or 35 or 36 or 37 or 38 or 39 41. 5 and 17 and 25 and 40	**Strategy:** Search: ((arthroplasty OR shoulder arthroplasty OR total shoulder arthroplasty OR anatomic OR hemiarthroplasty OR reverse total shoulder arthroplasty OR orthopedics OR orthopedic procedures OR osteoarthritis OR (osteoarthritis AND orthop*)) AND (artificial intelligence OR neural network OR deep learning OR artificial neural network OR machine learning OR machine intelligence OR algorithms) AND (predict* OR predictive value of test OR score OR scores OR scoring system* OR observ* OR observer variation OR detect* or evaluat* OR analy* OR assess* OR measure* OR area under curve OR ROC curve OR classification) AND (shoulder* OR glenohumeral* OR glenoid OR humer*)) AND ((“2024/11/02"[Date - Publication] : “3000"[Date - Publication]))	**Strategy:** 1. exp Shoulder/ or exp Shoulder Joint/ or shoulder*.mp. 2. glenohumeral*.mp. 3. exp Humerus/ or humer* 4. exp Glenoid cavity/ or glenoid.mp. 5. 1 or 2 or 3 or 4 6. exp Orthopedics/ or orthopedics.mp. 7. exp Orthopedic procedures/ or orthopedic procedures.mp. 8. orthop*.mp. 9. exp Arthroplasty, Replacement/ or exp Arthroplasty/ or arthroplasty.mp. or exp Arthroplasty, Replacement. Shoulder/ 10. shoulder arthroplasty.mp. 11. total shoulder arthroplasty.mp. 12. anatomic*.mp. 13. hemiarthroplasty.mp. or exp Hemiarthroplasty/ 14. reverse total shoulder arthroplasty.mp. 15. osteoarthr*.mp. or exp Osteoarthritis/ 16. 8 and 15 17. 6 or 7 or 8 or 9 or 10 or 11 or 12 or 13 or 14 or 15 or 16 18. exp Artificial Intelligence/ or artificial intelligence.mp. 19. exp Neural Networks, Computer/ or neural network.mp. 20. exp Machine Learning/ or machine learning.mp. 21. machine intelligence.mp. 22. exp Algorithms/ or algorithms.mp. 23. exp Deep Learning/ or deep learning.mp 24. artificial neural network.mp. 25. 18 or 19 or 20 or 21 or 22 or 23 or 24 26. predict*.mp. 27. predictive value of test.mp. or exp “Predictive Value of Tests"/ 28. score.mp. 29. scores.mp. 30. scoring system*.mp. 31. observ*.mp. 32. observer variation.mp. or exp Observer Variation/ 33. detect*.mp. 34. evaluat*.mp. 35. analy*.mp. 36. assess*.mp. 37. measure*.mp. 38. area under curve.mp. or exp Area Under Curve/ 39. ROC curve.mp. or exp ROC Curve/ 40. 26 or 27 or 28 or 29 or 30 or 31 or 32 or 33 or 34 or 35 or 36 or 37 or 38 or 39 41. 5 and 17 and 25 and 40
