# Supplementary material for: Transparency of reporting and methodological conduct of prognostic and diagnostic clinical prediction models developed using machine learning in total shoulder arthroplasty: A systematic review and critical appraisal
Source: Shoulder Elbow. 2026 Jan 27:17585732251412368. Online ahead of print. doi: 10.1177/17585732251412368 (PMC12846907; doi:10.1177/17585732251412368)
Supplement: sj-docx-1-sel-10.1177_17585732251412368 - Supplemental material for Transparency of reporting and methodological conduct of prognostic and diagnostic clinical prediction models developed using machine learning in total shoulder arthroplasty: A systematic review and critical appraisal [file sj-docx-1-sel-10.1177_17585732251412368.docx]

Appendix Table 1. Search strategy

| **EMBASE:** 1428 studies | **MEDLINE:** 637 studies | **PUBMED:** 21 studies | **COCHRANE:** 29 studies |
| --- | --- | --- | --- |
| **Strategy:**   1. exp Shoulder/ or exp Shoulder Joint/ or shoulder*.mp. 2. glenohumeral*.mp. 3. exp Humerus/ or humer* 4. exp Glenoid cavity/ or glenoid.mp. 5. 1 or 2 or 3 or 4 6. exp Orthopedics/ or orthopedics.mp. 7. exp Orthopedic procedures/ or orthopedic procedures.mp. 8. orthop*.mp. 9. exp Arthroplasty, Replacement/ or exp Arthroplasty/ or arthroplasty.mp. or exp Arthroplasty, Replacement. Shoulder/ 10. shoulder arthroplasty.mp. 11. total shoulder arthroplasty.mp. 12. anatomic*.mp. 13. hemiarthroplasty.mp. or exp Hemiarthroplasty/ 14. reverse total shoulder arthroplasty.mp. 15. osteoarthr*.mp. or exp Osteoarthritis/ 16. 8 and 15 17. 6 or 7 or 8 or 9 or 10 or 11 or 12 or 13 or 14 or 15 or 16 18. exp Artificial Intelligence/ or artificial intelligence.mp. 19. exp Neural Networks, Computer/ or neural network.mp. 20. exp Machine Learning/ or machine learning.mp. 21. machine intelligence.mp. 22. exp Algorithms/ or algorithms.mp. 23. exp Deep Learning/ or deep learning.mp 24. artificial neural network.mp. 25. 18 or 19 or 20 or 21 or 22 or 23 or 24 26. predict*.mp. 27. predictive value of test.mp. or exp "Predictive Value of Tests"/ 28. score.mp. 29. scores.mp. 30. scoring system*.mp. 31. observ*.mp. 32. observer variation.mp. or exp Observer Variation/ 33. detect*.mp. 34. evaluat*.mp. 35. analy*.mp. 36. assess*.mp. 37. measure*.mp. 38. area under curve.mp. or exp Area Under Curve/ 39. ROC curve.mp. or exp ROC Curve/ 40. 26 or 27 or 28 or 29 or 30 or 31 or 32 or 33 or 34 or 35 or 36 or 37 or 38 or 39 41. 5 and 17 and 25 and 40 | **Strategy:**   1. exp Shoulder/ or exp Shoulder Joint/ or shoulder*.mp. 2. glenohumeral*.mp. 3. exp Humerus/ or humer* 4. exp Glenoid cavity/ or glenoid.mp. 5. 1 or 2 or 3 or 4 6. exp Orthopedics/ or orthopedics.mp. 7. exp Orthopedic procedures/ or orthopedic procedures.mp. 8. orthop*.mp. 9. exp Arthroplasty, Replacement/ or exp Arthroplasty/ or arthroplasty.mp. or exp Arthroplasty, Replacement. Shoulder/ 10. shoulder arthroplasty.mp. 11. total shoulder arthroplasty.mp. 12. anatomic*.mp. 13. hemiarthroplasty.mp. or exp Hemiarthroplasty/ 14. reverse total shoulder arthroplasty.mp. 15. osteoarthr*.mp. or exp Osteoarthritis/ 16. 8 and 15 17. 6 or 7 or 8 or 9 or 10 or 11 or 12 or 13 or 14 or 15 or 16 18. exp Artificial Intelligence/ or artificial intelligence.mp. 19. exp Neural Networks, Computer/ or neural network.mp. 20. exp Machine Learning/ or machine learning.mp. 21. machine intelligence.mp. 22. exp Algorithms/ or algorithms.mp. 23. exp Deep Learning/ or deep learning.mp 24. artificial neural network.mp. 25. 18 or 19 or 20 or 21 or 22 or 23 or 24 26. predict*.mp. 27. predictive value of test.mp. or exp "Predictive Value of Tests"/ 28. score.mp. 29. scores.mp. 30. scoring system*.mp. 31. observ*.mp. 32. observer variation.mp. or exp Observer Variation/ 33. detect*.mp. 34. evaluat*.mp. 35. analy*.mp. 36. assess*.mp. 37. measure*.mp. 38. area under curve.mp. or exp Area Under Curve/ 39. ROC curve.mp. or exp ROC Curve/ 40. 26 or 27 or 28 or 29 or 30 or 31 or 32 or 33 or 34 or 35 or 36 or 37 or 38 or 39 41. 5 and 17 and 25 and 40 | **Strategy:**  Search: ((arthroplasty OR shoulder arthroplasty OR total shoulder arthroplasty OR anatomic OR hemiarthroplasty OR reverse total shoulder arthroplasty OR orthopedics OR orthopedic procedures OR osteoarthritis OR (osteoarthritis AND orthop*)) AND (artificial intelligence OR neural network OR deep learning OR artificial neural network OR machine learning OR machine intelligence OR algorithms) AND (predict* OR predictive value of test OR score OR scores OR scoring system* OR observ* OR observer variation OR detect* or evaluat* OR analy* OR assess* OR measure* OR area under curve OR ROC curve OR classification) AND (shoulder* OR glenohumeral* OR glenoid OR humer*)) AND (("2024/11/02"[Date - Publication] : "3000"[Date - Publication])) | **Strategy:**   1. exp Shoulder/ or exp Shoulder Joint/ or shoulder*.mp. 2. glenohumeral*.mp. 3. exp Humerus/ or humer* 4. exp Glenoid cavity/ or glenoid.mp. 5. 1 or 2 or 3 or 4 6. exp Orthopedics/ or orthopedics.mp. 7. exp Orthopedic procedures/ or orthopedic procedures.mp. 8. orthop*.mp. 9. exp Arthroplasty, Replacement/ or exp Arthroplasty/ or arthroplasty.mp. or exp Arthroplasty, Replacement. Shoulder/ 10. shoulder arthroplasty.mp. 11. total shoulder arthroplasty.mp. 12. anatomic*.mp. 13. hemiarthroplasty.mp. or exp Hemiarthroplasty/ 14. reverse total shoulder arthroplasty.mp. 15. osteoarthr*.mp. or exp Osteoarthritis/ 16. 8 and 15 17. 6 or 7 or 8 or 9 or 10 or 11 or 12 or 13 or 14 or 15 or 16 18. exp Artificial Intelligence/ or artificial intelligence.mp. 19. exp Neural Networks, Computer/ or neural network.mp. 20. exp Machine Learning/ or machine learning.mp. 21. machine intelligence.mp. 22. exp Algorithms/ or algorithms.mp. 23. exp Deep Learning/ or deep learning.mp 24. artificial neural network.mp. 25. 18 or 19 or 20 or 21 or 22 or 23 or 24 26. predict*.mp. 27. predictive value of test.mp. or exp "Predictive Value of Tests"/ 28. score.mp. 29. scores.mp. 30. scoring system*.mp. 31. observ*.mp. 32. observer variation.mp. or exp Observer Variation/ 33. detect*.mp. 34. evaluat*.mp. 35. analy*.mp. 36. assess*.mp. 37. measure*.mp. 38. area under curve.mp. or exp Area Under Curve/ 39. ROC curve.mp. or exp ROC Curve/ 40. 26 or 27 or 28 or 29 or 30 or 31 or 32 or 33 or 34 or 35 or 36 or 37 or 38 or 39 41. 5 and 17 and 25 and 40 |
