# Supplementary material for: Transparency of reporting and methodological conduct of prognostic and diagnostic clinical prediction models developed using machine learning in total shoulder arthroplasty: A systematic review and critical appraisal
Source: Shoulder Elbow. 2026 Jan 27:17585732251412368. Online ahead of print. doi: 10.1177/17585732251412368 (PMC12846907; doi:10.1177/17585732251412368)
Supplement: sj-docx-2-sel-10.1177_17585732251412368 - Supplemental material for Transparency of reporting and methodological conduct of prognostic and diagnostic clinical prediction models developed using machine learning in total shoulder arthroplasty: A systematic review and critical appraisal [file sj-docx-2-sel-10.1177_17585732251412368.docx]

Appendix Table II - Transparent Reporting of a Multivariable Prediction Model for Individual Prognosis or Diagnosis (TRIPOD) ratings for all included studies.

| **Study** | **Q**  **4a** | **Q**  **4b** | **Q**  **5a** | **Q**  **5b** | **Q**  **6a** | **Q**  **6b** | **Q**  **7a** | **Q**  **7b** | **Q**  **9** | **Q**  **10a** | **Q**  **10b** | **Q**  **10d** | **Q**  **11** | **Q**  **13a** | **Q**  **13b** | **Q**  **14a** | **Q**  **14b** | **Q**  **15a** | **Q**  **15b** | **Q**  **16** |
| --- | --- | --- | --- | --- | --- | --- | --- | --- | --- | --- | --- | --- | --- | --- | --- | --- | --- | --- | --- | --- |
| Gowd AK, 2019 | Yes | Yes | Yes | Yes | Yes | No | Yes | No | Yes | No | Yes | Yes | NA | No | No | Yes | NA | No | No | Yes |
| Biron DR, 2020 | Yes | Yes | Yes | Yes | Yes | NA | Yes | NA | Yes | Yes | Yes | Yes | NA | Yes | Yes | Yes | Yes | No | No | Yes |
| Kumar V, 2020 | Yes | Yes | No | Yes | Yes | NA | Yes | No | Yes | No | Yes | Yes | NA | No | No | Yes | Yes | No | No | Yes |
| Kumar V, 2021 | Yes | Yes | No | Yes | Yes | NA | No | NA | Yes | No | Yes | Yes | Yes | No | No | Yes | NA | No | No | Yes |
| Arvind V, 2021 | Yes | Yes | Yes | Yes | Yes | Yes | Yes | No | No | No | Yes | Yes | N/A | No | No | Yes | Yes | No | No | Yes |
| Polce E, 2021 | Yes | Yes | Yes | Yes | Yes | NA | Yes | NA | Yes | No | Yes | Yes | NA | No | Yes | No | NA | Yes | No | Yes |
| Karnuta JM, 2020 | Yes | Yes | Yes | Yes | Yes | No | Yes | No | No | No | Yes | Yes | NA | Yes | Yes | Yes | Yes | Yes | No | Yes |
| Kumar V, 2022 | Yes | Yes | No | Yes | Yes | NA | No | NA | Yes | No | Yes | Yes | NA | No | No | Yes | NA | No | No | Yes |
| Devana SK, 2021 | Yes | Yes | Yes | Yes | Yes | No | No | No | No | No | Yes | Yes | NA | No | No | Yes | Yes | Yes | No | Yes |
| Lopez CD, 2021 | Yes | Yes | No | Yes | Yes | NA | No | NA | Yes | Yes | Yes | Yes | NA | No | No | Yes | Yes | No | No | Yes |
| McLendon PB, 2021 | Yes | Yes | No | Yes | Yes | No | Yes | No | Yes | Yes | Yes | Yes | NA | No | No | Yes | Yes | Yes | No | Yes |
| Devana SK, 2022 | Yes | Yes | No | Yes | Yes | NA | Yes | No | No | No | Yes | Yes | NA | No | No | Yes | NA | No | No | Yes |
| Kumar V, 2021 | Yes | Yes | Yes | Yes | Yes | No | Yes | No | Yes | Yes | Yes | Yes | NA | No | No | No | No | No | No | No |
| Lopez CD, 2022 | Yes | Yes | No | Yes | Yes | NA | Yes | NA | Yes | Yes | Yes | Yes | NA | No | No | Yes | Yes | No | No | Yes |
| Kumar V, 2022 | Yes | Yes | Yes | Yes | Yes | No | Yes | No | Yes | Yes | Yes | Yes | NA | No | No | No | No | No | No | Yes |
| Gowd AK, 2022 | Yes | No | No | Yes | Yes | NA | Yes | Yes | No | No | Yes | Yes | No | No | No | Yes | NA | No | No | Yes |
| Oeding JK, 2023 | Yes | Yes | Yes | Yes | No | Yes | yes | No | Yes | Yes | Yes | Yes | NA | Yes | Yes | Yes | No | No | No | Yes |
| Schneller T, 2024 | Yes | Yes | Yes | Yes | Yes | No | Yes | No | Yes | Yes | Yes | Yes | N/a | Yes | Yes | Yes | No | No | No | No |
| Franceschetti E, 2024 | Yes | Yes | Yes | Yes | Yes | No | Yes | No | Yes | Yes | Yes | Yes | NA | Yes | Yes | Yes | No | No | No | Yes |
| Kim A, 2024 | Yes | Yes | Yes | Yes | Yes | No | Yes | No | Yes | Yes | Yes | Yes | NA | Yes | Yes | Yes | No | No | No | Yes |
| Miltenberg B, 2024 | Yes | Yes | Yes | Yes | Yes | No | Yes | No | Yes | Yes | Yes | Yes | NA | Yes | Yes | Yes | Yes | No | No | Yes |
| Marigi EM, 2025 | Yes | Yes | Yes | Yes | Yes | No | Yes | No | Yes | Yes | Yes | Yes | NA | Yes | Yes | Yes | No | No | No | No |
| Parmigiani O, 2025 | Yes | Yes | Yes | Yes | Yes | No | Yes | No | Yes | Yes | Yes | Yes | NA | Yes | Yes | Yes | No | No | No | No |
| Powell CM, 2025 | Yes | Yes | Yes | Yes | Yes | No | Yes | No | Yes | Yes | Yes | Yes | NA | Yes | Yes | Yes | No | No | No | No |
