# Supplementary material for: Transparency of reporting and methodological conduct of prognostic and diagnostic clinical prediction models developed using machine learning in total shoulder arthroplasty: A systematic review and critical appraisal
Source: Shoulder Elbow. 2026 Jan 27:17585732251412368. Online ahead of print. doi: 10.1177/17585732251412368 (PMC12846907; doi:10.1177/17585732251412368)
Supplement: sj-docx-3-sel-10.1177_17585732251412368 - Supplemental material for Transparency of reporting and methodological conduct of prognostic and diagnostic clinical prediction models developed using machine learning in total shoulder arthroplasty: A systematic review and critical appraisal [file sj-docx-3-sel-10.1177_17585732251412368.docx]

Appendix Table III - Prediction Model Risk of Bias Assessment Tool (PROBAST) ratings for all included studies.

| Study | Participants | Predictors | Outcomes | Analysis | Participants | Predictors | Outcomes | ROB | Applicability |
| --- | --- | --- | --- | --- | --- | --- | --- | --- | --- |
| Gowd 2019 | (-) | (?) | (?) | (-) | (-) | (-) | (-) | (?) | (-) |
| Biron 2020 | (-) | (?) | (-) | (+) | (-) | (-) | (-) | (+) | (-) |
| Kumar 2020 | (-) | (?) | (?) | (+) | (-) | (-) | (-) | (+) | (-) |
| Kumar 2021 | (-) | (+) | (+) | (+) | (-) | (-) | (-) | (+) | (-) |
| Arvind 2021 | (-) | (-) | (-) | (+) | (-) | (-) | (-) | (+) | (-) |
| Polce 2021 | (-) | (?) | (?) | (?) | (-) | (-) | (-) | (?) | (-) |
| Karnuta 2020 | (-) | (+) | (-) | (+) | (-) | (?) | (-) | (+) | (-) |
| Kumar 2022 | (-) | (?) | (-) | (+) | (-) | (-) | (-) | (+) | (-) |
| Devana 2021 | (-) | (?) | (?) | (+) | (-) | (-) | (?) | (+) | (?) |
| Lopez 2021 | (+) | (-) | (?) | (?) | (-) | (-) | (-) | (+) | (-) |
| McLendon 2021 | (+) | (-) | (-) | (+) | (-) | (-) | (?) | (+) | (?) |
| Devana 2022 | (-) | (-) | (?) | (?) | (-) | (-) | (-) | (?) | (-) |
| Kumar 2021 | (?) | (?) | (+) | (?) | (-) | (?) | (-) | (+) | (?) |
| Lopez 2022 | (-) | (-) | (?) | (-) | (-) | (-) | (?) | (?) | (?) |
| Kumar 2022 | (?) | (?) | (+) | (?) | (-) | (-) | (-) | (+) | (-) |
| Gowd 2022 | (-) | (-) | (?) | (+) | (-) | (-) | (?) | (+) | (?) |
| Oeding JF 2023 | (-) | (-) | (-) | (-) | (-) | (-) | (-) | (-) | (-) |
| Schneller T 2024 | (-) | (-) | (?) | (+) | (-) | (-) | (?) | (+) | (?) |
| Franceschetti E 2024 | (-) | (?) | (-) | (+) | (-) | (-) | (-) | (+) | (-) |
| Kim A 2024 | (-) | (-) | (-) | (-) | (-) | (-) | (-) | (-) | (-) |
| Miltenberg B 2024 | (-) | (-) | (-) | (-) | (-) | (-) | (-) | (-) | (-) |
| Marigi EM 2025 | (-) | (-) | (-) | (-) | (-) | (-) | (-) | (-) | (-) |
| Parmigiani O 2025 | (-) | (+) | (-) | (?) | (-) | (-) | (-) | (+) | (-) |
| Powell CM 2025 | (-) | (-) | (?) | (+) | (-) | (-) | (?) | (+) | (?) |

(-) Low Risk

(+) High Risk

(?) Unclear Risk
